# Monoterpenoid Indole Alkaloids from *Alstonia rupestris* with Cytotoxic, Anti-Inflammatory and Antifungal Activities

**DOI:** 10.3390/molecules18067309

**Published:** 2013-06-21

**Authors:** Wei Wang, Ming-He Cheng, Xiao-Hua Wang

**Affiliations:** 1 Department of Pharmacy, No. 455 Hospital of People’s Liberation Army, Shanghai 200052, China; 2 Department of Pharmacology, School of Pharmacy, Second Military Medical University, Shanghai 200433, China; 3 Department of Pharmacy, No. 202 Hospital of People’s Liberation Army, Shenyang 110003, Liaoning, China

**Keywords:** *Alstonia rupestris*, Apocynaceae, monoterpenoid indole alkaloids, cytotoxicity, anti-inflammatory, antifungal

## Abstract

Phytochemical investigation of the 70% EtOH extract of the leaves of *Alstonia scholaris* afforded seven new monoterpenoid indole alkaloids: scholarisins I-VII (**1-7**), and three known compounds: (3*R*,5*S*,7*R*,15*R*,16*R*,19*E*)-scholarisine F (**8**), 3-*epi*-dihydro- corymine (**9**), and (*E*)-16-formyl-5α-methoxystrictamine (**10**). Structural elucidation of all the compounds was accomplished by spectral methods such as 1D- and 2D-NMR, IR, UV, and HRESIMS. The isolated compounds were tested *in vitro* for cytotoxicity against seven tumor cell lines, anti-inflammatory activities against Cox-1 and Cox-2, and antifungal potential against five species of fungi. Compounds **1**, **6**, and **10** exhibited significant cytotoxicities against all the tested tumor cell lines with IC_50_ values of less than 30 μM and selective inhibition of Cox-2 comparable with the standard drug NS-398 (>90%). Additionally, **1**, **2**, **3** and **8** showed antifungal activity against two fungal strains (*G. pulicaris* and *C. nicotianae*).

## 1. Introduction

**Figure 1:**
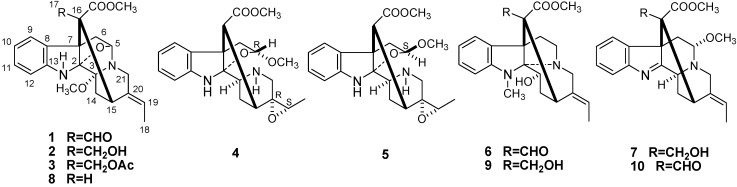
The structures of compounds **1**–**10**.

The genus *Alstonia*, which belongs to the family Apocynaceae, is widely distributed throughout the tropical areas of the World, including Central America, Africa, Indo-Malaya, Australia and Asia [1,2,3]. The genus *Alstonia* comprises about 60 species, eight of which grow in China [4]. Several of these species are used in Traditional Chinese Medicine, for example in the treatment of malaria, dysentery, defervescence, antitussive, and to arrest hemorrhages [5,6,7,8,9,10]. Monoterpenoid indole alkaloids occur abundantly in the family Apocynaceae [11,12,13,14,15,16,17], and to date, more than 300 such monoterpenoid indole alkaloids have been reported from the plants of this genus [18,19,20,21,22]. This type of alkaloids originates from the condensation of tryptophan with secologanin to give strictosidine and then further elaboration gives an impressive array of structural variants [23]. Monoterpenoid indole alkaloids were reported to have anticancer, antibacterial, antifertility, and anti-tussive activities [24,25,26,27,28]. *Alstonia rupestris* Kerr. is usually endemic in the western part of Guangxi Province of China. To the best of our knowledge, the phytochemistry of the *A. rupestris* has been rarely reported previously, which prompted the present study. Present investigation on chemical constituents of the EtOH extract of the leaves of *A. rupestris* led to seven new monoterpenoid indole alkaloids: scholarisin I-VII (**1**–**7**) together with three known compounds: (3*R*,5*S*,7*R*,15*R*,16*R*,19*E*)-scholarisine F (**8**), 3-*epi*-dihydrocorymine (**9**), and (*E*)-16- formyl-5α-methoxystrictamine (**10**) (Figure 1). The structures of these compounds were elucidated mainly by NMR spectroscopic and mass spectroscopic methods. Furthermore, all the alkaloids were *in vitro* evaluated for their cytotoxic, anti-inflammatory and antifungal activities.

## 2. Results and Discussion

**Figure 2:**
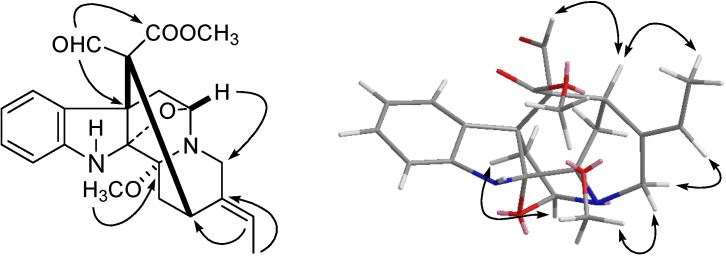
Key HMBC (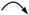) and NOESY (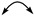) correlations of of compound **1**.

Compound **1** was obtained as a white amorphous powder. The positive HRESIMS spectrum displayed a pseudomolecular ion at *m/z* 419.1585 [M+Na]^+^ (calcd for C_22_H_24_N_2_O_5_Na, 419.1583) consistent with a molecular formula of C_22_H_24_N_2_O_5_, corresponding to 12 degrees of unsaturation. Its UV characteristic absorption peaks at 285, 240, and 228 nm was for a indole chromophore. The IR spectrum exhibited absorptions at 3,425 and 1,725 cm^−1^ for NH and C=O functions, respectively. Its ^13^C-NMR spectrum showed 22 carbon signals [OCH_3_ × 2, CH_3_ × 1, CH_2_ (sp^3^) × 3, CH (sp^3^) × 2, C (sp^3^) × 4, CH (sp^2^) × 6 and C (sp^2^) × 4, Table 1]. The ^1^H-NMR spectrum exhibited four aromatic proton signals [*δ*_H_ 7.31 and 6.68 (each, 1H, dd, *J* = 7.8, 1.8 Hz), 6.84 and 7.08 (each, 1H, dt, *J* = 7.8, 1.8 Hz)] ascribed to an *ortho*-disubstituted benzene ring, an ethylidene side chain [1.51 (d, *J* = 7.0, H-18) and 5.47 (q, *J* = 7.0, H-19)], a NH signal at *δ*_H_ 4.96, a downfield proton signal at *δ*_H_ 8.52 due to a formyl group and a singlet peak at *δ*_H_ 3.50 assigned to one methoxy group. The HMBCs of the proton signal at *δ*_H_ 3.50 (OCH_3_) with the carbon signals at *δ_C_* 85.4 (C-3) indicated the methoxy group substitution at C-3. In the NOE spectrum, the correlation of the methoxy group at C-3 with H-21*α* (*δ*_H_ 2.26) indicated the *α* orientation of the methoxy group (Figure 2). The NOE correlation of H-5/H-6*β* and H-15/H-17 evidenced the *β* and *α* orientation of H-5 and H-15, respectively. The *E*-form of the double bond of 19/20 was determined on the basis of the NOE correlations of H-19/21 and H-18/15. These data suggested that the structure of **1** was almost identical with (3*R*,5*S*,7*R*,15*R*,16*R*,17*R*,19*E*)-scholarisine F (**8**) [28]. The distinct difference was the presence of one more formyl group at C-16 in **1**, which was supported by the observation of the HMBC correlations of the proton signal at *δ*_H_ 8.52 (H-17) with the carbon signals of C-7, C-15, and carbonyl group of carbomethoxy and the downfield chemical shift of C-16 from *δ_C_* 51.5 in **8** to *δ_C_* 65.6 in **1**. Thus, **1** was named as scholarisin I and the structure was showed in Figure 1.

**Table 1:** ^13^C-NMR data of compounds **1**–**7** in CDCl_3_.

No.	1	2	3	4	5	6	7
2	107.5	105.6	107.0	103.5	104.9	102.0	186.7
3	85.4	85.1	85.3	51.9	51.1	70.6	50.8
5	86.1	85.6	85.9	105.4	106.9	63.5	89.7
6	42.5	44.4	42.9	42.8	40.9	42.5	43.6
7	55.1	53.1	55.5	52.3	52.4	62.4	53.2
8	131.5	131.6	133.1	138.0	136.8	131.3	142.4
9	126.0	126.0	126.1	122.7	123.4	121.6	121.6
10	121.5	122.1	121.9	119.9	120.0	127.5	124.9
11	128.9	128.6	128.9	128.1	128.5	130.6	125.4
12	110.7	110.4	110.3	109.1	110.2	112.5	120.8
13	147.9	148.3	147.8	144.6	145.9	148.7	156.1
14	25.0	28.5	23.6	26.2	27.0	34.2	35.8
15	34.1	33.5	37.3	27.7	28.3	34.2	33.4
16	65.6	54.4	55.0	52.8	52.1	65.6	58.8
17	197.4	65.9	66.2	-	-	194.5	63.0
18	13.4	13.7	13.6	12.8	12.9	15.1	13.5
19	120.7	119.7	119.8	58.4	58.5	133.2	127.8
20	130.5	131.3	131.3	61.5	61.6	129.9	137.7
21	47.9	48.1	48.2	44.7	44.5	66.6	50.5
CO_2_CH_3_	168.0	169.3	169.4	172.1	172.6	171.9	169.1
CO_2_CH_3_	52.7	51.5	51.7	51.8	51.6	53.9	51.4
*N*_1_-CH_3_	-	-	-	-	-	50.5	-
OCH_3_	48.6	48.5	48.4	54.8	57.0	-	54.7
COCH_3_	-	-	171.3	-	-	-	-
COCH_3_	-	-	20.1	-	-	-	-

Compound **2** was isolated as a white amorphous powder. Its positive HRESIMS spectrum showed a quasimolecular ion peak at *m/z* 399.1924 [M+H]^+^, consistent with the molecular formula C_22_H_26_N_2_O_5_. Comparing the ^1^H- and ^13^C-NMR data of **2** with those of compound **1**, the data were almost identical. The only significant difference was that the formyl group at C-16 was replaced by a hydroxymethyl group in compound **2**, which was confirmed by the HMBC correlations of the proton signal of the hydroxymethyl group [*δ*_H_ 3.22, 3.46 (each, 1H, d, *J* = 13.8)] with the carbonyl group of carbomethoxy at *δ_C_* 169.3. On the basis of the observation of NOESY data similar to those of **1**, the stereochemistry of **2** was expected to be the same. Accordingly, the structure of **2** was established as scholarisin II and the structure was showed in Figure 1.

Compound **3** was obtained as a white amorphous powder. The EIMS afforded a molecular weight of *m/z* 440, and its HRESIMS revealed the [M+H]^+^ peak at *m/z* 441.2025 (calcd. for C_24_H_29_N_2_O_6_. 441.2026), corresponding to the molecular formula C_24_H_28_N_2_O_6_. The general features of its IR and NMR spectra closely resembled those of **2**, except for the presence of one more Ac group. The OAc group were positioned at C-17 based on HMBC correlations of H-17 [*δ* 3.87 and 4.01 (each, 1H, d, *J* = 13.8)] with the acyl carbon (*δ* 171.3) of the acetyl group. The stereochemistry of **3** was expected to be the same as **2** on the basis of the NOESY data. Thus, compound **3** was elucidated as scholarisin III and the structure was as shown in Figure 1.

**Figure 3:**
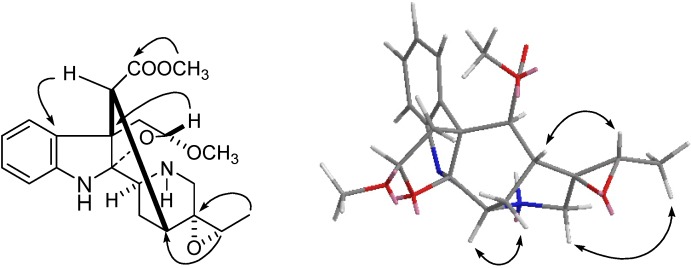
Key HMBC (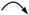) and NOESY (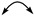) correlations of of compound **4**.

Compound **4**, a white amorphous powder, gave one quasimolecular ion peak at *m/z* 409.1735 [M+Na]^+^ in its HRESIMS, accounting for a molecular formula of C_21_H_26_N_2_O_5_. The IR spectrum showed absorption peaks at 3430 (NH) and 1740 (C=O) cm^−1^. In the ^1^H-NMR spectrum, four aromatic proton signals at *δ*_H_ 7.12 and 6.56 (each, 1H, dd, *J* = 8.2, 1.8 Hz), 6.78 and 7.06 (each, 1H, dt, *J* = 8.2, 1.8 Hz)] showed an *ortho*-disubstituted benzene ring, two singlet peaks at *δ*_H_ 3.10 and 3.70 were assigned to the protons of a methoxy and a carbomethoxy group, respectively. Its ^13^C-NMR displayed a pattern similar to that of scholarisine C [28], except that the double bond of 19/20 was substituted by a methine at *δ_C_* 58.4 (d, C-19) and a quaternary carbon at *δ_C_* 61.5 (s, C-20). The molecular formula C_21_H_26_N_2_O_5_ displayed 10 unsaturation degrees, which indicated the presence of 19,20-epoxide combined with the appropriate NMR data. The NOE correlation of H-3/H-14*α* indicated the *α* configuration of C-3 (Figure 3). On the basis of the NOE correlations of H-15/H-19 and H-18/21, the configurations of C-19 and C-20 was elucidated as *S* and *R*, respectively. The *R* configuration of C-5 was determined by the coupling constant of H-5 (d, *J* = 5.2 Hz) compared with that of scholarisine C (d, *J* = 5.4 Hz) [28]. Therefore, compound **4** was determined as scholarisin IV, with the structure as shown in Figure 1.

Compound **5**, a white amorphous powder, exhibited a molecular formula of C_21_H_26_N_2_O_5_, based on the HRESIMS spectrum which showed a pseudomolecular ion at *m/z* 387.1923 [M+H]^+^ (calcd. 387.1920). The general features of NMR spectra closely resembled those of **4,** except for the configuration of C-5. H-5 was observed as a doublet of doublets at *δ*_H_ 4.90 (1H, dd, *J* = 7.2, 5.6 Hz) in the ^1^H-NMR spectrum, which indicated the *S* configuration of C-5 [28]. This evidence indicated that compound **5** was an isomer of **4**, and **5** was identified as scholarisin V, with the structure shown in Figure 1.

**Figure 4:**
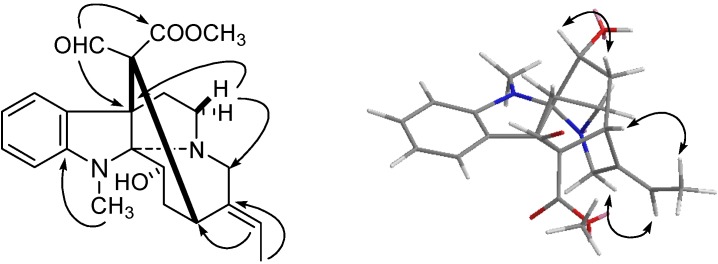
Key HMBC (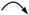) and NOESY (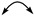) correlations of of compound **6**.

Compound **6**, a white amorphous powder, exhibited a molecular formula of C_22_H_26_N_2_O_4_, based on the HRESIMS spectrum which showed a pseudomolecular ion at *m/z* 383.1974 [M+H]^+^ (calcd. 383.1971). The UV absorptions at 286, 241 and 220 nm showed the presence of an indole chromophore. The IR spectrum indicated the presence of formyl group (1720 cm^−1^), and benzene ring (1650 cm^−1^). Its ^13^C-NMR spectrum showed 22 carbon signals [CH_3_ × 2, OCH_3_ × 1, CH_2_ (sp^3^) × 4, CH (sp^3^) × 3, C (sp^3^) × 2, CH (sp^2^) × 6 and C (sp^2^) × 4, Table 1]. The ^1^H-NMR spectrum exhibited four *ortho*-disubstituted aromatic proton signals [*δ*_H_ 7.76 and 6.79 (each, 1H, dd, *J* = 7.8, 2.0 Hz), 6.92 and 7.24 (each, 1H, dt, *J* = 7.8, 2.0 Hz)], an ethylidene side chain [1.83 (d, *J* = 7.2, H-18) and 5.86 (q, *J* = 7.2, H-19)], a *N*-CH_3_ (*δ*_H_ 2.03), a formyl group (*δ*_H_ 8.55), one methoxy group (*δ*_H_ 3.80). These data showed similarities to those of 3-*epi*-dihydrocorymine (**9**) [29]. Comparing the ^1^H- and ^13^C-NMR data of **6** with those of 3-*epi*-dihydrocorymine, the data were almost identical. The only significant difference was that the signals of one hydroxymethyl group was replaced by those of the formyl group (*δ*_C_ 194.5; *δ*_H_ 8.55), which was supported by the observation of the HMBC correlations of the proton signal at *δ*_H_ 8.55 (H-17) with the carbon signals of C-7, C-15 and carbonyl group of carbomethoxy (Figure 4). In the NOE experiment, the correlation of H-3/H-21*β* indicated the *β* orientation of C-3. The *E*-form of the double bond of 19/20 was determined on the basis of the NOE correlations of H-19/21 and H-18/15. On the basis of the observation of NOESY data similar to those of **9**, the stereochemistry of **6** was expected to be the same. Accordingly, the structure of **6** was established as scholarisin VI (Figure 1).

**Figure 5:**
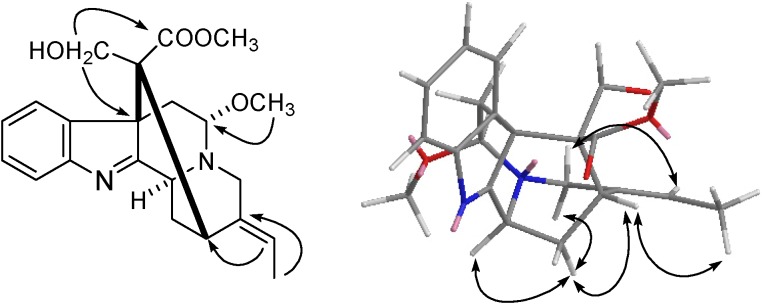
Key HMBC (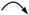) and NOESY (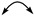) correlations of of compound **7**.

The molecular formula of compound **7** was assigned as C_22_H_26_N_2_O_4_ on the basis of the quasi- molecular ion peak [M+Na]^+^ at *m/z* 405.1787 in the HRESIMS. The ^13^C-NMR and DEPT spectra displayed signals of three Me, four CH_2_, and nine CH groups, together with six quaternary C-atoms. The NMR signals at *δ*_H_ 7.79 (dd, *J* = 8.0, 2.0 Hz, H-9), 7.32 (dt, *J* = 8.0, 2.0 Hz, H-10), 7.14 (dt, *J* = 8.0, 2.0 Hz, H-11), 7.39 (dd, , *J* = 8.0, 2.0 Hz, H-12), and those at *δ*_C_ 142.4 (C-8), 121.6 (C-9), 124.9 (C-10), 125.4 (C-11) , 120.8 (C-12), and 156.1 (C-13) were characteristic for the presence of an indole moiety. The NMR data of **7** was almost identical with those of (*E*)-16-formyl-5*α*-methoxystrictamine (**10**) [30]. The only significant difference was that a hydroxymethyl group [*δ*_H_ 3.68, 3.91 (each, 1H, d, *J* = 13.2)] in **7** instead of the formyl group at C-16 in **10**, was confirmed by HMBC correlations of H-17 with C-7, C-15 and carbonyl group of carbomethoxy (Figure 5). The relative configuration of compound **7** was determined by the NOESY experiment. Based on the similarity of NOE spectrum with that of **10**, the NOE interactions of H-3/H-14*α*, H-15/H-14*α*, H-5/H-21, and H-15/H-18 indicated the H-3*α*, H-15*α*, and 19 *E* configuration compared with that of (*E*)-16-formyl-5*α*-methoxystrictamine. From these data, **7** was named scholarisin VII.

The *in vitro* cytotoxic activities of the isolated alkaloids were evaluated against seven tumor cell lines by using the revised MTT method as described in the Experimental. The results are summarized in Table 2. Alkaloids **1**, **6**, and **10** exhibited significant cytotoxicity (IC_50_ < 30 μM) while **2**, **3**, and **7**–**9** showed weak cytotoxic activites (IC_50_ > 40 μM) against all the tested tumor cell lines. Furthermore, alkaloids **4** and **5** without the linkage between C-5 and N-4 were non-cytotoxic (IC_50_ > 80 μM). The results indicated that the linkage between C-5 and N-4 was essential for cytotoxic properties, while the formyl group on C-16 might strengthen the cytotoxic activities for this type of alkaloids.

**Table 2:** Cytotoxicity of compounds **1**–**10** against seven human tumor cell lines (IC_50_, μM) ^a^.

Compound	Cell lines						
	A-549	BGC-823	HepG2	HL-60	MCF-7	SMMC-7721	W480
**1**	10.3	11.3	9.2	12.0	10.7	23.7	28.0
**2**	52.7	61.8	49.0	59.4	54.3	59.7	59.5
**3**	44.1	40.8	30.4	39.6	36.8	47.0	40.1
**4**	-	-	-	-	-	-	94.9
**5**	97.4	-	-	-	-	92.1	-
**6**	13.0	12.9	10.8	12.3	11.3	24.9	29.9
**7**	49.1	53.2	43.6	48.2	46.7	49.4	52.7
**8**	47.8	51.5	44.8	50.7	48.9	53.2	51.4
**9**	61.3	67.1	58.3	71.8	64.2	66.2	62.1
**10**	16.1	15.7	14.8	17.2	14.7	31.2	35.5
Doxorubicin	18.3	14.7	22.0	31.7	24.9	35.4	15.9

^a^ Doxorubicin activities are expressed as IC_50_ values in nM, and those of compounds **1**–**8** are expressed as IC_50_ values in μM. (-) IC_50_ > 100 μM.

The compounds **1**–**10** were tested *in vitro* for their anti-inflammatory activities. The results of the anti-inflammatory assay were summarized in Table 3. Among the assayed compounds, only alkaloids **1**, **6** and **10** with formyl group at C-16 displayed selective inhibition of Cox-2 (> 90%). Alkaloids **2**–**5** and **7**–**9** had no anti-inflammatory activities or selective inhibition of Cox-2 comparable to those of **1**, **6** and **10** although they possess the same monoterpene indole skeleton. The observations indicated that the formyl group at C-16 should be essential for this type of alkaloids to possess the anti-inflammatory activity.

**Table 3:** Evaluation of Anti-Inflammatory Activity of Compounds **1**–**10** ^a^.

Compound	COX-1	COX-2
**1**	45.2	96.4
**2**	<0	14.9
**3**	12.9	50.4
**4**	<0	21.1
**5**	<0	24.3
**6**	36.9	95.5
**7**	<0	17.6
**8**	<0	<0
**9**	13.6	46.8
**10**	38.5	92.0
SC-560	63.2	
NS-398		97.1

^a^ Percent inhibition (all compounds and reference drugs concentration: 100 μM).

All compounds were tested for their antifungal activities by the disc diffusion method by measuring the inhibition zones and for the most active compounds, minimum inhibitory concentration (MIC) values were also determined. Antifungal properties (Table 4) showed that alkaloids **1**, **2**, **3** and **8** exhibited antifungal activity against two fungi (*G. pulicaris* and *C. nicotianae*), with MIC values of 0.64–0.69 mM, 1.37–1.44 mM, 1.80–1.91 mM and 1.55–1.71 mM, respectively. Alkaloids **1** possessed rather higher antifungal potent with lower MIC value. The other alkaloids had no a antifungal activities. These result suggested that the structure skeleton of **1** may be essential and the *N*-carbamate group could strengthen the antifungal activities of this type of alkaloids.

**Table 4:** Antifungal activities (zones of inhibition/and MIC mM, n = 3) of compounds **1**–**10**.

Compound	*G. pulicaris*	*A. alternata*	*C. nicotianae*	*P. capsici.*	*G. amomi*
1	20/0.69	-	19/0.64	-	-
2	18/1.37	-	17/1.44	-	-
3	15/1.91	-	16/1.80	-	-
4	-	-	-	-	-
5	-	-	-	-	-
6	-	-	-	-	-
7	-	-	-	-	-
8	15/1.71	-	17/1.55	-	-
9	-	-	-	-	-
10	-	-	-	-	-
Nystatin	21/0.007	19/0.006	19/0.006	20/0.010	19/0.009

-: No activity.

## 3. Experimental

### 3.1. General

Optical rotations were determined with a JASCO P2000 digital polarimeter (Tokyo, Japan). Ultraviolet (UV) and infrared (IR) spectra were obtained on JASCO V-650 and JASCO FT/IR-4100 spectrophotometers (Tokyo, Japan), respectively. NMR spectra were measured on a Bruker AM-600 spectrometer (^1^H-NMR) and Bruker AM-400 spectrometer (^13^C-NMR). EIMS and HREIMS (70 eV) were carried out on a Finnigan MAT 95 mass spectrometer. All solvents used were of analytical grade (Shanghai Chemical Reagents Company Ltd., Shanghai, China). Silica gel (200–300 mesh), silica gel H (Qingdao Haiyang Chemical Co. Ltd., Qingdao, China), C18 reversed-phase silica gel (150–200 mesh, Merck), and MCI gel (CHP20P, 75–150 lm, Mitsubishi Chemical Industries Ltd., Tokyo, Japan) were used for column chromatography. HPLC separation was performed on an instrument consisting of a Waters 600 controller, a Waters 600 pump, and a Waters 2487 dual λ absorbance detector, with a Prevail (250 × 10 mm i.d.) preparative column packed with C18 silica (5 μm).

### 3.2. Plant Material

The leaves of *A. scholaris* were collected in Yongning, Guangxi Province, China, in September 2011. The sample was identified by one of the authors (G. B. Shi). A specimen (201109001AS) was deposited in the Herbarium of Shengyang Medicine College, Shengyang, China.

### 3.3. Extraction and Isolation

The dried leaves of *A. scholaris* (16 kg) were powdered and extracted thrice with 70% ethanol (25 L) at room temperature and then concentrated under reduced pressure to give a crude extract (198.5 g). The crude extract was partitioned between equal volumes of ethyl acetate and water to provide an EtOAc-soluble (77.5 g) and an aqueous layer. The EtOAc-soluble fraction was subjected to silica gel column chromatography eluted with CHCl_3_/MeOH (from 100:1 to 1:1) to yield seven fractions (F1-F7). F2 (4.3 g) was further subjected to silica gel column chromatography eluted with CHCl_3_/MeOH (from 10:1 to 1:1) to give three subfractions F2a (276 mg), F2b (253 mg), and F2c (226 mg). Subfraction F2a was separated by repeated column chromatography over Sephadex LH-20 (CHCl_3_/MeOH, 1:1, and MeOH), then puried on silica gel column chromatography eluted with n-hexane/EtOAc (7:3) to yield **8** (99.1 mg). Subfraction F2b was further subjected to reverse phase high performance liquid chromatography (RP-HPLC) eluted with methanol/water (70:30) to furnish compounds **4** (56.1 mg) and **5** (56.1 mg). F2c was subjected to a normal phase high performance liquid chromatography (NP-HPLC) eluted with n-hexane/ethyl acetate (8:1) to afford compound **3** (65.3 mg) and **1** (64.3 mg). F3 (4.9 g) was subjected to a silica gel column chromatography eluted with n-hexane/EtOAc (from 100% n-hexane to 100% EtOAc) to furnish four subfractions (F3a-F3d). F3b (588 mg) was separated on a reverse-phase HPLC eluted with methanol/water (65:35) to yield three compounds **2** (59.3 mg) and **10** (49.5 mg). F3c (406 mg) was chromatographed on a reverse phase HPLC column eluted with methanol/water (55:45) to yield **7** (78.9 mg). F4 (2.3 g) was separated using a silica gel column and eluted with *n*-hexane/ CH_2_Cl_2_/MeOH (30:70:1.5) to give two subfractions F4a (380 mg) and F4b (260 mg). F4a was further subjected to a reverse phase HPLC column eluted with methanol/water (65:35) to provide two compounds **6** (69.3 mg) and **9** (49.5 mg).

*Scholarisin I* (**1**). White amorphous powder. ${\left[\alpha \right]}_{D}^{23.3}$: −38.8 (*c* = 0.80, MeOH). UV (CHCl_3_) λ_max_ (log *ε*) 285 (2.81), 240 (3.40), 228 (2.83) nm. IR (KBr) *ν_max_* 3425, 1725, 1465, 1175, 1090, 1062, 870, 753 cm^−1^. ^1^H-NMR (CDCl_3_, 600 MHz) and ^13^C-NMR (CDCl_3_, 125 MHz) data see Table 5 and Table 1 respectively. EI-MS *m/z*: 396 ([M]^+^). HRESIMS (pos.) *m/z*: 419.1585 ([M+Na]^+^, C_22_H_24_N_2_O_5_Na. calc. 419.1583).

*Scholarisin II* (**2**). White amorphous powder. ${\left[\alpha \right]}_{D}^{23.3}$: −30.9 (*c* = 0.62, MeOH). UV (CHCl_3_) λ_max_(log*ε*) 286 (2.85), 240 (3.46), 228 (2.78), 228 (2.82) nm. IR (KBr) *ν_max_* 3445, 3420, 1730, 1464, 1170, 1065, 875 cm^−1^. ^1^H-NMR (CDCl_3_, 600 MHz) and ^13^C-NMR (CDCl_3_, 125 MHz) data see Table 5 and Table 1 respectively. EI-MS *m/z*: 398 ([M]^+^). HRESIMS (pos.) *m/z*: calc. 399.1924 [M+H]^+^, C_22_H_27_N_2_O_5_. calc. 399.1920).

*Scholarisin III* (**3**). White amorphous powder. ${\left[\alpha \right]}_{D}^{23.3}$: −35.4 (*c* = 0.76, MeOH). UV (CHCl_3_) λ_max_(log*ε*) 286 (2.80), 242 (3.30), 227 (2.89) nm. IR (KBr) *ν_max_* 3425, 1735, 1460, 1172, 1092, 1062, 871 cm^−1^. ^1^H-NMR (CDCl_3_, 600 MHz) and ^13^C-NMR (CDCl_3_, 125 MHz) data see Table 5 and Table 1 respectively. EI-MS *m/z*: 440 ([M]^+^). HRESIMS (pos.) *m/z*: 441.2025 ([M+H]^+^, C_24_H_29_N_2_O_6_. calc. 441.2026).

*Scholarisin IV* (**4**). White amorphous powder. ${\left[\alpha \right]}_{D}^{23.3}$: −38.2 (*c* = 0.30, MeOH). UV (CHCl_3_) λ_max_(log*ε*) 284 (3.45), 241 (3.62), 220 (3.32) nm. IR (KBr) *ν_max_* 3430, 2950, 1740, 1628, 1465, 1102, 750 cm^−1^. ^1^H-NMR (CDCl_3_, 600 MHz) and ^13^C-NMR (CDCl_3_, 125 MHz) data see Table 5 and Table 1 respectively. EI-MS: 386 ([M]^+^). HRESIMS (pos.) *m/z*: 409.1735 ([M+Na]^+^, C_21_H_26_N_2_O_5_Na. calc. 409.1739).

*Scholarisin V* (**5**). White amorphous powder. ${\left[\alpha \right]}_{D}^{23.3}$: −18.5 (*c* = 0.23, MeOH). UV (CHCl_3_) λ_max_(log*ε*) 285 (3.15), 240 (3.68), 228 (3.01) nm. IR (KBr) *ν_max_* 3423, 1735, 1635, 1465, 1447, 1170, 1035 cm^−1^. ^1^H-NMR (CDCl_3_, 600 MHz) and^13^C-NMR (CDCl_3_, 125 MHz) data see Table 5 and Table 1 respectively. EI-MS *m/z*: 386 ([M]^+^). HRESIMS (pos.) *m/z*: 387.1923 ([M+H]^+^, C_21_H_27_N_2_O_5_. calc. 387.1920).

*Scholarisin VI* (**6**). White amorphous powder. ${\left[\alpha \right]}_{D}^{23.3}$: −38.5 (*c* = 0.35, MeOH). UV (CHCl_3_) λ_max_(log*ε*) 286 (3.30), 241 (3.81), 220 (3.31) nm. IR (KBr) *ν_max_* 3428, 1720, 1650, 1605, 1465, 1215, 1165, 1035 cm^−1^. ^1^H-NMR (CDCl_3_, 600 MHz) and ^13^C-NMR (CDCl_3_, 125 MHz) data see Table 5 and Table 1 respectively. EI-MS *m/z*: 382 ([M]^+^). HRESIMS (pos.) *m/z*: 383.1974 ([M+H]^+^, C_22_H_27_N_2_O_4_. calc. 383.1971).

*Scholarisin VII* (**7**). White amorphous powder. ${\left[\alpha \right]}_{D}^{23.3}$: −56.5 (*c* = 0.19, MeOH). UV (CHCl_3_) λ_max_(log*ε*) 285 (3.41), 239 (3.73), 218 (3.27), 195 (3.83) nm. IR (KBr) *ν_max_* 3448, 1735, 1635, 1455, 1442, 1166, 1015 cm^−1^. ^1^H-NMR (CDCl_3_, 600 MHz) and ^13^C-NMR (CDCl_3_, 125 MHz) data see Table 2 and Table 1 respectively. EI-MS *m/z*: 382 ([M]^+^). HRESIMS (pos.) *m/z*: 405.1787 ([M+Na]^+^, C_22_H_26_N_2_O_4_Na. calc. 405.1790).

**Table 5:** ^1^H-NMR data of compounds **1**–**7** in CDCl_3_ (*δ* in ppm and *J* in Hz).

No.	1	2	3	4	5	6	7
*N*_1_-H	4.96 (s)	4.98 (s)	5.00 (s)	5.04 (s)	4.43 (s)	-	-
3	-	-	-	3.96 (m)	3.36 (m)	4.73 (dd, 14.0,4.0)	4.48 (dd, 14.0,4.0)
5*α*	-	-	-	-	-	2.18 (m)	-
5*β*	4.88 (dd, 4.2,3.6)	4.86 (d, 5.2)	4.92 (dd, 4.0,3.6)	5.28 (dd, 4.0,3.8)	4.92 (dd, 7.2,5.6)	2.32 (m)	3.84 (dd, 4.0,3.8)
6*α*	2.25 (dd, 13.6,4.2)	2.26 (d, 13.8)	2.29 (d, 14.0)	2.75 (dd, 14.0,4.0)	2.76 (dd, 14.4,5.6)	2.06 (m)	2.20 (dd, 14.0,4.0)
6*β*	3.41 (dd, 13.6,3.6)	3.48 (dd, 13.8,5.2)	3.52 (dd, 14.0,5.2)	3.08 (dd, 14.0,3.8)	3.10 (dd, 14.4,7.2)	2.33 (m)	3.75 (dd, 14.0,3.8)
9	7.31 (dd, 7.8,1.8)	7.72 (dd, 8.2,2.0)	7.76 (dd, 7.8,2.0)	7.12 (dd, 8.2,1.8)	7.14 (dd, 8.0,2.0)	7.76 (dd, 7.8,2.0)	7.79 (dd, 8.0,2.0)
10	6.84 (dt, 7.8,1.8)	6.70 (dt, 8.2,2.0)	6.82 (dt, 7.8,2.0)	6.78 (dt, 8.2,1.8)	6.77 (dt, 8.0,2.0)	6.92 (dt, 7.8,2.0)	7.39 (dt, 8.0,2.0)
11	7.08 (dt, 7.8,1.8)	6.98 (dt, 8.2,2.0)	7.14 (dt, 7.8,2.0)	7.06 (dt, 8.2,1.8)	7.10 (dt, 8.0,2.0)	7.24 (dt, 7.8,2.0)	7.14 (dt, 8.0,2.0)
12	6.68 (dd, 7.8,1.8)	6.66 (dd, 8.2,2.0)	6.78 (dd, 7.8,2.0)	6.56 (dd, 8.2,1.8)	6.66 (dd, 8.0,2.0)	6.79 (dd, 7.8,2.0)	7.32 (dd, 8.0,2.0)
14*α*	2.26 (dd, 14.0,3.8)	2.30 (dd, 14.0,3.8)	2.33 (dd, 13.8,3.8)	2.23 (m)	2.25 (m)	2.27 (m)	2.88 (m)
14*β*	2.18 (dd, 14.0,4.0)	2.27 (dd, 14.0,4.0)	2.29 (dd, 13.8,4.0)	1.91 (m)	1.93 (m)	2.24 (m)	1.96 (m)
15	3.63 (dd, 4.0,3.8)	3.69 (dd, 4.0,3.8)	3.72 (dd, 4.0,3.8)	2.95 (dd, 4.0,3.8)	2.97 (dd, 4.0,3.8)	3.62 (dd, 4.0,3.8)	3.66 (dd, 4.0,3.8)
16	-	-	-	2.92 (d, 4.8)	2.59 (d, 4.2)	-	-
17a	8.52 (s)	3.32 (d, 13.8)	3.87 (d, 13.6)	-	-	8.55 (s)	3.68 (d, 13.2)
17b	-	3.46 (d, 13.8)	4.01 (d, 13.6)	-	-	-	3.91 (d, 13.2)
18	1.51 (d, 7.0)	1.55 (d, 7.2)	1.58 (d, 7.0)	1.40 (d, 7.0)	1.41 (d, 6.8)	1.83 (d, 7.2)	1.55(d, 7.0)
19	5.47 (q, 7.0)	5.48 (q, 7.2)	5.50 (q, 7.0)	2.93 (q, 7.0)	2.95 (q, 6.8)	5.86 (d, 7.2)	5.54(d, 7.0)
21*α*	3.85 (d, 13.8)	3.87 (d, 14.2)	3.89 (d, 13.8)	3.36 (d, 14.0)	3.39 (d, 14.0)	3.88 (d, 13.8)	4.07 (d, 13.6)
21*β*	3.31 (d, 13.8)	3.33 (d, 14.2)	3.35 (d, 13.8)	3.08 (d, 14.0)	3.11 (d, 14.0)	3.35 (d, 13.8)	3.08 (d, 13.6)
CO_2_*CH_3_*	3.69 (s)	3.78 (s)	3.82 (s)	3.70 (s)	3.67 (s)	3.80 (s)	3.71 (s)
*N*_1_-CH_3_	-	-	-	-		2.03 (s)	-
OCH_3_	3.50 (s)	3.52 (s)	3.53 (s)	3.10 (s)	3.41 (s)	-	3.25 (s)
CO*CH_3_*	-	-	1.53 (s)	-		-	-

### 3.4. Cytotoxicity Assay *in Vitro*

The isolated compounds 1–10 were subjected to cytotoxic evaluation against A-549 cells (lung cancer), BGC-823 cells (human gastric carcinoma), HepG2 cells (human hepatocellular carcinoma), HL-60 (human myeloid leukemia), MCF-7 cells (human breast cancer), SMMC-7721 (hepatocellular carcinoma), and W480 (colon cancer) by employing the revised MTT method as described in the literature [31]. Doxorubicin was used as the positive control. All tumor cell lines were cultured on RPMI-1640 medium supplemented with 10% fetal bovine serum, 100 U mL^−1^ penicillin and 100 μg/mL streptomycin in 25 cm^3^ culture flasks at 37 °C in humidified atmosphere with 5% CO_2_. For the cytotoxicity tests, cells in exponential growth stage were harvested from culture by trypsin digestion and centrifuging at 180 ×*g* for 3 min, then resuspended in fresh medium at a cell density of 5 × 10^4^ cells per mL. The cell suspension was dispensed into a 96-well microplate at 100 μL per well, and incubated in humidified atmosphere with 5% CO_2_ at 37 °C for 24 h, and then treated with the compounds at various concentrations (0, 1, 10, 100 μM). After 48 h of treatment, 50 μL of 1 mg/mL MTT solution was added to each well, and further incubated for 4 h. The cells in each well were then solubilized with DMSO (100 μL for each well) and the optical density (OD) was recorded at 570 nm. All drug doses were tested in triplicate and the IC_50_ values were derived from the mean OD values of the triplicate tests *versus* drug concentration curves. The 50% inhibition concentration (IC_50_ value) was determined by curve fitting and was used as criteria to judge the cytotoxicity (active: IC_50_ ≤ 20 μM; moderately active: 20 μM < IC_50_ ≤ 80 μM; not active: IC_50_ > 80 μM). All cell lines were purchased from Cell Bank of Shanghai Institute of Biochemistry & Cell Biology, Chinese Academy of Sciences. Other reagents were purchased from Shanghai Sangon Biological Engineering Technology & Services Co., Ltd. (Shanghai, China).

### 3.5. Anti-Inflammatory Assay *in Vitro*

The anti-inflammatory activities were determined according to a literature method with minor modifications [32]. The reaction system was incubated at 25 °C for 5 min, by sequential addition of the buffer, heme, test compounds, and Cox-1 or Cox-2 into the system followed by mixing with TMPD and arachidonic acid. The absorbance value was recorded at a wavelength of 590 nm after another 15 min of incubation at 25 °C. SC-560 and NS-398 were used as positive controls, which gave the inhibition of Cox-1 (63.20%) and Cox-2 (97.13%) respectively (Table 3). All cell lines were purchased from the Cell Bank of Shanghai Institute of Biochemistry & Cell Biology, Chinese Academy of Sciences. (Shanghai, China).

### 3.6. Antifungal Activity Bioassay

All compounds (purity > 90%) were screened for their antifungal activity *in vitro* using the disk-diffusion method as described in the literature with minor modifications [33]. Strains including five species of fungi [*Gibberella pulicaris* (KZN 4207), *Alternaria alternata* (TX-8025), *Colletotrichum nicotianae* (SACC-1922), *Phytophthora capsici* (KACC-40157), *Gonatopyricularia amomi* (MB-9671)] were used. Nystatin were used as positive controls for antifungal activity. A disk containing only DMSO was used as the negative control. Agar medium was used in the antifungal activity. To each agar plate, an inoculum containing 10^7^ bacteria/mL or a 0.5 optical density of the McFarland Scale was incorporated. The plates were solidified and sterile filter paper disks (6-mm diameter) were done on each one. Solution of each compound (5 mM) in DMSO, antifungal agents (nystatin 10 μM/mL), and control vehicles (DMSO) were added into too. The plates were aerobically incubated at 37 °C for the five species of fungi during 24 h. The diameter of the inhibition zone was measured for testing of antifungal activities. Experiments were performed in triplicate, and the results are presented as the mean values of the diameters of the inhibitory zones from three runs. The MIC values of the most active compounds, in the previous experiment, were determined using the dilution method in 96-hole plates. The diameters of the inhibitory zones and the MIC value were used as criteria to judge the antimicrobial activity (active: the diameters of the inhibitory zones ≥ 16 mm, MIC ≤ 5 mM; moderately active: the diameters of the inhibitory zones are visible, MIC > 5 mM; not active: the diameters of the inhibitory zones are invisible). All fungal were purchased from the Shanghai Institute of Biochemistry & Cell Biology, Chinese Academy of Sciences (Shanghai, China).

## 4. Conclusions

A chemical investigation of the 70% EtOH extract of the dried leaves of *A. scholaris* resulted in the isolation of seven new monoterpenoid indole alkaloids: scholarisin I-VII (**1-7**), and three known compounds: (3*R*,5*S*,7*R*,15*R*,16*R*,19*E*)-scholarisine F (**8**), 3-*epi*-dihydrocorymine (**9**), and (*E*)-16- formyl-5α-methoxystrictamine (**10**). All the isolated compounds 1-10 were evaluated for their cytotoxic activities against seven tumor cell lines and alkaloids **1**, **6** and **10** possessed significant cytotoxicities against all the tested tumor cell lines with low IC_50_ values (<30 μM). In screening *in vitro* of cytotoxic activities of all the alkaloids anti-inflammatory properties against Cox-1 and Cox-2, **1**, **6** and **10** showed selective inhibition of Cox-2 (>90%) comparable with the standard drug NS-398. Additionally, **1**, **2**, **3** and **8** had antifungal activity against two fungal spp. (*G. pulicaris* and *C. nicotianae*).
